# INTERNET USE AND LONELINESS OF OLDER ADULTS OVER TIME: THE MEDIATION EFFECT OF SOCIAL CONTACT

**DOI:** 10.1093/geroni/igz038.697

**Published:** 2019-11-08

**Authors:** Kexin Yu, Kexin Yu, Shinyi Wu, Iris Chi

**Affiliations:** University of Southern California, Los Angeles, California, United States

## Abstract

Internet is increasingly popular among older adults and have changed interpersonal interactions. However, it remains controversial whether older people are more or less lonely with internet use. This paper tests the longitudinal association of internet use and loneliness among older people. One pathway that explains the association, the mediation effect of social contact, was examined. Data from the 2006, 2010 and 2014 waves of Health and Retirement Study was used. Hierarchical liner modeling results showed internet use was related to decreased loneliness over 12-year period of time (b=-0.044, p<.001). Internet use was associated with more social contact with family and friends overtime (b=0.261, p<.001), social contact was related to less perceived loneliness longitudinally (b=0.097, p<.001). The total effect of internet use on loneliness is -0.054 and the mediated effect is -0.025. The findings imply that online activities can be effective for reducing loneliness for older people through increased social contact.

